# The Impact on Nursing Students of Creating Audiovisual Material through Digital Storytelling as a Teaching Method

**DOI:** 10.3390/ijerph18020694

**Published:** 2021-01-15

**Authors:** Julián Rodríguez-Almagro, María del Carmen Prado-Laguna, Antonio Hernández-Martínez, Adrián Monzón-Ferrer, Juan Carlos Muñoz-Camargo, Mairena Martín-Lopez

**Affiliations:** 1 Department of Nursing, Physiotherapy and Occupational Therapy, Ciudad Real Faculty of Nursing, Universidad de Castilla-La Mancha, 13071 Ciudad Real, Spain; Julianj.rodriguez@uclm.es (J.R.-A.); Carmina.Prado@uclm.es (M.d.C.P.-L.); Adrian.Monzon@uclm.es (A.M.-F.); JuanCarlos.Munoz@uclm.es (J.C.M.-C.); Mairena.Martin@uclm.es (M.M.-L.); 2 Department of Inorganic, Organic and Biochemical Chemistry, Regional Center of Biomedical Research (CRIB), Universidad de Castilla-La Mancha, 13071 Ciudad Real, Spain; 3 Department of Inorganic, Organic and Biochemical Chemistry, Faculty of Chemical Sciences and Technologies, Universidad de Castilla-La Mancha, 13071 Ciudad Real, Spain

**Keywords:** video teaching, nursing students, students, teaching, university, digital storytelling

## Abstract

The creation of videos in teaching has a high educational potential and is a challenge that can motivate students. There is little evidence on the use of this method when applied to the creation of digital stories. Thus, the aim of this study was to measure student satisfaction with the creation of audiovisual material through digital storytelling, measure its usefulness, and evaluate its impact on their motivation to study the subject. As a secondary objective, we intended to determine the influence of this learning experience on raising awareness of society toward mental illnesses by measuring the impact by the number of views on social networks. A cross-sectional descriptive study design was used. The participants were 90 third-year nursing students enrolled in the subject “Psychiatric Nursing”. The students created eight themed videos (depression, suicide, anxiety, anorexia, mobile phone addiction, obsessive-compulsive disorder, drug addiction, schizophrenia). The students were then asked to complete an ad hoc questionnaire on the matter. A total of 90% of the nursing students thought that creating the videos improved the acquisition of nursing knowledge, 91.2% replied that they would like to use the method in other subjects on the degree in nursing syllabus, and 67.8% thought that their clinical skills improved after using narrated digital stories to create videos. Students acknowledged that this training activity helped them feel better prepared and helped them better understand the subject. They believe that this teaching technique is more stimulating and more enjoyable than the conventional system, giving them more motivation to study the subject. Students acknowledge that the experience gained from this initiative has helped them feel better prepared and helped them better understand the subject, and they think that it will be a useful resource in the future as it has improved the process of the creation of audiovisual material through digital storytelling.

## 1. Introduction

The traditional model of higher education (HE) is evolving. Students are becoming powerful consumers of education and demand up-to-date teaching and support models [1]. In this regard, multiple authors recommend that healthcare educators should update their traditional teaching methods to match the current state of technology [1,2,3,4].

Although Kirschner and De Bruychere [5] state that there is a lot of evidence to show that digital natives do not exist and that, although students from this generation have experienced a connected digital world, they are often unable to deal with modern technology in the way that has been ascribed to them. The same authors also go on to say that this idea raises the question as to how education can or should be redesigned so that effective, efficient, and enjoyable use is made of the tools and technologies available—with their concomitant pedagogies—in a digital and connected world [5].

Currently, students have developed new communication channels in which comprehension occurs through images such as television, video games, and the Internet. When it comes to audiovisual teaching methods and new technologies, video stands out. It is becoming an attractive option for many educators, especially in higher education, where the use of video and multimedia content is expanding [6,7,8]. Mayer defines multimedia learning as learning in which the subject constructs mental representations from a multimedia presentation, that is, in which the subject constructs knowledge [9].

The use of audiovisual material for teaching and learning is an interesting topic for both researchers and educators. However, teaching strategies for using this material as a resource that is fully integrated into the classroom have yet to be exploited. Despite this, we can say that this technology is highly available to teachers and students, and it is possible to obtain products with a reasonable level of quality [6,8].

Educational videos are a very effective method if correctly applied to the context of the class and if they have meaningful elements that show a direct relationship between their content, the syllabus, and those teaching it. [10,11] 

Some of the main advantages of using video are improved psychomotor development and knowledge acquisition [12,13,14]. Another advantage is its low cost, especially when it can reach a large audience (better benefit–cost ratio). Furthermore, videos can be viewed over and over again, and can be used either individually or collectively [15].

Currently, multiple authors have begun using video in education as a new training tool called “digital storytelling” [16,17]. Digital storytelling is the idea of telling a story, often with a strong emotional component, through the use of a variety of digital multimedia such as images, audio, music, video, and the narrator’s voice [17].

Digital storytelling can be especially useful in teaching nursing, as one significant reported benefit of digital storytelling is the way it can provide authentic learning experiences, thus improving the development of professional identity and critical thought [17,18]. According to several authors [19,20] digital storytelling done for educational purposes is very highly valued by nursing students [16].

While storytelling in generic education or management settings is focused on communicating the organization’s policies and values, nursing training differs in that there is a demand for stories that provide information on healthcare experiences. It is therefore appropriate to consider how digital storytelling has been used both to attract service users and to transmit messages about health [21].

Christiansen [19] observed that creating digital stories about patients/illnesses could improve transformative learning, while Stacey and Hardy [20] found that digital storytelling by recently graduated nursing students provided them with valuable information about what to expect when entering clinical practice. In a recent study by Wood and Paiadelis [22], students created their own digital stories to encourage reflection on clinical practice studies.

Building on the benefits described by Charon [23], digital storytelling is used in nursing education to describe the experiences of registered nurses and students, as a way of exploring or reflecting on the realities of clinical practice [21]. Digital storytelling has also been used to promote reflection by nursing students [20] and to develop empathy and understanding among health professionals [24].

In summary, we believe that the creation of educational videos in a university setting may have high potential due to its applicability and its ability to motivate students. These videos can be especially useful when used in digital storytelling in subjects like psychiatry and mental health. However, there is currently very limited evidence about how useful they are.

Nursing is a discipline that can exert a significant social influence as a health educator. In this regard, creating narrated video stories could be a very useful tool to teach and raise awareness among the population, making nursing a vector for the transfer of knowledge to society.

To make digital storytelling by students into a useful pedagogical tool for teaching nursing, more knowledge is needed about the experiences of nursing students. 

## 2. Material and Methods

### 2.1. Study Design and Sample Selection

A descriptive, transversal, quantitative interventional study was carried out. The study population was composed of nursing students from the University of Ciudad Real (Spain). The sampling was intentional including students enrolled in the subject “Psychiatry and Mental Health” (*n* = 90) from the third year of the degree in nursing and who voluntarily took part in the video teaching activity (*n* = 90). All of the students were from the same level, the third year of the nursing degree.

### 2.2. Rules and Design of the Creation of Audiovisual Material through Digital Storytelling for Nursing Students

In 2017, the School of Nursing of XXXXXXXX approached the University of XXXXXX, the organization we belong to, with the educational innovation project “*En cualquier momento a cualquier persona*” [Anyone, Any Time], created by the school’s teaching staff, involving the whole center in the implementation of the same.

The project had two main objectives, framed within the subject Psychiatry and Mental Health. First, a teaching objective based on teaching students about mental illnesses. The students themselves would be responsible for creating audiovisual material in the form of narrated digital stories for future years, through a collaborative experience based on designing, producing, and disseminating videos. This material could be used by students in future years as part of the teaching material provided to them, and could also be used as a complement to lectures.

The second objective was to transfer social knowledge to the general public. The videos were made to raise public awareness of mental illnesses with the objective of reducing social stigma.

Students created audiovisual material in various classrooms at the School of Nursing, where students had attended lectures in the subject Psychiatry and Mental Health. This subject is included in the second semester of the third year of the degree in nursing, lasts 12 weeks, and includes 40 h of theory and 100 h of theoretical-practical classes.

The following steps were taken to create the audiovisual material:

1. The students were introduced to the topic through lectures and practical seminars. They were then divided into eight large groups of 11 to 12 students, as indicated in the subject academic guide for this type of activity. Using their creativity and expression, they created various narrated digital stories discussing these illnesses respectfully and from a scientific point of view, as a starting point for raising public awareness about mental illnesses.
2. Before recording the audiovisual material, each group performed a literature search to complement the topic they had been randomly assigned to create a digital story about. They also watched several examples to motivate them to create their own digital stories.
3. The topics were depression, suicide, anxiety, anorexia, mobile phone addiction, obsessive-compulsive disorder, drug addiction, and schizophrenia.
4. The students familiarized themselves with the official University of XXXXXXX’s YouTube channel in order to then be able to upload their videos and store them on the channel for future consultations by all students, thereby renewing the teaching and learning process. The channel can be accessed at https://www.youtube.com/user/UCLMvideos.
5. They then created a webmix (on Symbaloo) for better interoperability and management of the videos uploaded to the YouTube channel. Additionally, through this platform or personal learning environment, documents can be added to complement the information in the videos, aiding understanding of these topics. In fact, each video is accompanied by an information sheet made by the students to complement the audiovisual material on each of the illnesses studied. The webmix can be accessed at https://www.symbaloo.com/mix/enfermeriacr1.
6. The students used Symbaloo to organize teaching resources created by students, with the approval of the professor. Symbaloo is a personal learning environment (PLE) that is fully integrable with the virtual platform Moodle in the UCLM Virtual Campus.
7. The students created the school’s official Twitter profile in order to disseminate the audiovisual material. The Twitter profile can be found at https://twitter.com/CR_Enfermeria.
8. The students then recorded their audiovisual material. To do this, they used the cameras on their own mobile phones, as they all had high-definition recording in a panoramic 16:9 format. The maximum duration of the videos was 6 min. They were also told that the videos could have English subtitles to reach a much wider audience. The only video with English subtitles was the one on anorexia, while the videos on anxiety and obsessive-compulsive disorder were subtitled in Spanish to cater to people with hearing problems. Furthermore, during the recording of each video, the same professor (who was also the lead investigator and the one teaching the subject) was always present to respond to any questions in relation to the topics being studied and to correct any errors in relation to the topics assigned to each group.
9. The school’s teaching staff reviewed the audiovisual material.
10. The videos were disseminated through information channels like Facebook, Twitter, YouTube, and Symbaloo.
11. An official video library was created, accessible from anywhere in the world, with the possibility of creating videos in English or with subtitles in English.
12. Final evaluation of the methodology used through anonymous, voluntary questionnaires.

On World Mental Health Day on 10 October 2018, the campaign “*En cualquier momento a cualquier persona*” [Anyone, Any Time] was launched as the final step of the entire project, which was very well received by the media, scientific associations, and the general public. The degree of participation was high from all those involved including mental health associations throughout Spain.

### 2.3. Study Variables and Data Collection

The study data were collected using an ad hoc online questionnaire created on the Moodle platform for the subject. On the last day of recording of the audiovisual material, an ad hoc questionnaire was distributed. Seven days later, all the students involved were sent an email reminding them to complete the questionnaire. To prevent students from responding more than once, only one response per Moodle platform user was allowed.

The sociodemographic variables ‘sex’ and ‘age’ were collected. They were asked three questions in relation to clinical rotations in mental health services and if they had any family members or friends with mental health problems. As outcome variables, and based on a questionnaire used in similar previous studies [25,26], the responses to the thirteen questions were assessed on a 5-point Likert-type scale ranging from 1 = strongly disagree to 5 = strongly agree, as follows:

1. I thought making the videos was easy.
2. The instructions for making the videos were easy to understand.
3. The instructions for accessing the videos once they had been made were easy to understand.
4. Making the videos has motivated me to learn about mental health problems.
5. I think that the experience of making the videos was very positive.
6. Being able to watch the videos at any time was very convenient and useful for improving subject knowledge.
7. I think that making the videos has improved my acquisition of mental health knowledge.
8. I have learnt clinical skills through using the videos.
9. I feel more prepared for clinical practice after making the videos.
10. I think I will use the videos to review my clinical skills in the future.
11. I would like the videos to be used more often in the teaching of clinical skills.
12. After making the videos, I have more empathy for people with mental health problems.
13. After making the videos, I am no longer afraid of dealing with people with mental health problems.

The study data were collected in October 2018 and were analyzed using the SPSS v. 24 statistics package (IBM, Armonk, NY, USA). In terms of the descriptive statistics, absolute and relative frequencies were used for the qualitative variables as well as the median and interquartile range for the Likert-type variables. The mean and standard deviation were used for the quantitative variables. To determine the reliability of the questionnaire used, Cronbach’s alpha was computed for the questionnaire as a whole and then after removing each item individually. The overall Cronbach’s alpha for the questionnaire was 0.866. The lowest Cronbach’s alpha after removing each item individually was 0.842.

### 2.4. Ethical Considerations

Participation in the study was voluntary. Groups of students who voluntarily agreed to be recorded gave their signed informed consent for the use of those images to be included in the videos.

The study was approved by the research committee of the University (IRB number: 24032017). Aside from the in-person explanation, the questionnaire included a summary of the study purpose and informed consent.

## 3. Results

### 3.1. Audiovisual Material Created

A total of eight themed videos were created. They were disseminated on social networks under the slogan “*En cualquier momento a cualquier persona*” [Anyone, Any Time].

The videos can be found at the following links:

Depression: https://youtu.be/BdPru8RSC4w
Suicide: https://youtu.be/xuiPBZcp7tQ
Anxiety: https://youtu.be/-SaqoOtfxe0
Anorexia: https://youtu.be/xPg24ydaAZk
Mobile phone addiction: https://youtu.be/UOOWZbtIANI
Obsessive-compulsive disorder: https://youtu.be/aBTOkopxaZc
Drug addiction: https://youtu.be/ZIdv7b5hT0o
Schizophrenia: https://youtu.be/xuiPBZcp7tQ

### 3.2. Media Impact of the Project

The videos were viewed more than 4500 times in the months following the date they were disseminated, with the subjects of suicide and anxiety receiving the most views.

The project was also featured several times in the local and regional press and on the official website of the University of Castilla-La Mancha. The professor responsible for the study was also interviewed by Healthcare Creators, a national digital media outlet focusing on healthcare-related content, which can be watched in full at the following link: https://youtu.be/vaiB-Zr4btA.

Finally, all of the material was integrated into Moodle within the subject Psychiatry and Mental Health. Moodle is the platform usually used by students to keep up to date with their subjects and download the necessary course material.

### 3.3. Evaluation of the Student Opinions about the Experience

The mean age of the participants was 21.8 years. A total of 86.7% (*n* = 78) were women and 13.3% (*n* = 12) were men; 48.9% said they had a family member with mental health problems and 34.4% said they had a friend with mental health problems.

The students gave median scores of 4.5 and 5 to the items regarding technical complexity. This means that they agreed that the videos were easy to make, and strongly agreed that the instructions for creating and accessing the videos were easy to understand.

They were then asked about aspects related to experience and satisfaction. The items about motivation and satisfaction both obtained median scores of 5 (strongly agree), while the students gave a median score of 4 (agree) to the item “I would like the videos to be used more often in the teaching of clinical skills”.

The next set of questions made reference to the perceived usefulness of this learning tool when it came to acquiring knowledge and improving skills. Here, the students gave median scores of 4 (agree) to the items about usefulness for improving subject knowledge, knowledge acquisition, and learning clinical skills. The lowest scores were observed in relation to the usefulness of the video for preparing for clinical practice and their future careers, with median scores of 3 (neutral).

Finally, the students were asked two questions about the reduction of mental health stigma. In this case, the students gave a median score of 5 (strongly agree) to the item about videos increasing their empathy toward people with mental health problems and a median score of 4 (agree) to the statement that the videos had reduced their fear of dealing with people with mental health problems. The rest of the information is shown in Table 1.

## 4. Discussion

The aim of this paper was to analyze the nursing students’ opinions and study motivations after creating audiovisual material through digital storytelling in university nursing studies. Students acknowledged that the experience gained from this initiative has helped them feel better prepared and helped them better understand the subject. They also thought that it will be a useful resource in the future, as it has increased their empathy and awareness of mental health problems and has improved the process of learning to use technology tools.

After analyzing our results, and in line with Forbes [27], we believe that the use of videos to teach clinical skills in nursing can be a promising teaching initiative to follow, but we must improve and document the current situation in this area of research. Our findings show that the students that participated in this project were very satisfied and confident, similar to what occurred in the sample in Herron’s study [28].

It is true that some items received low scores such as whether students felt better prepared for their everyday clinical practice after making the audiovisual material, but we must take into account that some students may lack abilities or interest in using social media as part of learning [2]. Balakrishnan [29] pointed out that there are incentives and barriers that influence the use of social networks and that ease of use was important to students that found them academically beneficial.

We therefore agree that learning activities that involve social networks and the creation of audiovisual material could be included in nursing education to develop digital professionalism, as suggested by Jones et al. [30].

Like Coyne [31], we believe, based on our results, that opportunities for visual and interactive learning like digital storytelling could become one of the students’ preferred learning systems. We also believe that videos should be used within a blended learning model to engage students and satisfy their learning needs.

It seems that narrated digital stories in this context offer an opportunity for students to empathize with people with illnesses and give them an idea of what can be a difficult transition from the relatively safe and predictable world of university to the apparent chaos of clinical practice. This can allow them to identify personal strategies to better cope with this transition period [20].

We think that the use of narrated digital stories by students, using multimedia technology for reflection, could make a significant contribution to learning in nursing education. The capacity to create and share authentic learning experiences and good content, along with a strong emphasis on stories, seems to get students highly involved, giving them more opportunities for reflection and learning by implementing the digital stories they themselves have created as a standard learning activity in nursing studies. Furthermore, as pointed out by Kristin et al. [16], it is an activity that does not require many additional resources due to the availability of the videos online, thus improving the student’s digital skills and tools.

It is vital for educators in nursing to develop active teaching strategies to encourage and improve clinical reasoning among nursing students [28]. It has been demonstrated that active and experiential learning techniques improve nursing students’ clinical reasoning and their capacity to apply knowledge to patient care in different situations [28,32,33].

### 4.1. Other Implications of Using Video Teaching in Nursing

This teaching experience may be applicable to other subjects and other types of teacher training outside of a university setting. Involving students in creating audiovisual material through narrated digital stories is a way of empathizing with patients and trying to understand their feelings, experiences, and perceptions. These types of experiences can be easily extrapolated to other public awareness campaigns, for example, on gender violence or the social inclusion of immigrants, refugees, etc.

However, the study also provides important information on the use of multimedia technology by students creating narrated digital stories, which could convey a more personal message, increasing the risk of placing the student in a vulnerable position. We therefore believe that these teaching strategies must be implemented in a safe, respectful environment, as mentioned in previous studies [16].

### 4.2. Study Limitations

This study presents certain limitations. Although the study sample was larger than previous studies in this field, we believe that it is still quite small due to the limited number of students enrolled in the subject. Another limitation is that there is no validated tool to determine satisfaction and perceived usefulness of this type of learning tool. We therefore had to create our own questionnaire, as was done in previous studies [25,26].

A further limitation is the idea of intrinsic motivation, as indicated by Ryan and Deci’s [34] self-determination theory, according to which someone that is motivated will be more productive than someone who is not. This may have biased our results.

### 4.3. Future Lines of Research and Implications for Practical Uses

A proposal to continue the project or develop a new project in the future would involve the use of this type of methodology in other subjects on the degree in nursing syllabus. More specifically, we would determine the effectiveness of this way of creating audiovisual material in these new subjects, and the impact on student’s teamwork. It would also be useful to evaluate the opinions of other professors in relation to this type of activity in the discipline of nursing, and whether they have a positive view of the use of these techniques as a source of knowledge generation and as a way of strengthening acquired knowledge by making use of different methods to improve learning. Greater contribution to the activity by students may result in improved skills and improved professional development.

## 5. Conclusions

Making educational videos through the use of narrated digital stories is a good teaching and learning strategy to reinforce the main themed units in nursing studies.

Students acknowledge that the experience gained from this initiative has helped them feel better prepared and helped them better understand the subject, and they think that it will be a useful resource in the future as it has increased their empathy and awareness of mental health problems and improved the process of learning to use technology tools.

## Figures and Tables

**Table 1:** Student assessment of the training activity.

Item	Level of Agreement					Median (IQR)
	Strongly Disagree (1 Point) *n* (%)	Disagree (2 Points) *n* (%)	Neutral (3 Points) *n* (%)	Agree (4 Points) *n* (%)	Strongly Agree (5 Points) *n* (%)	
**Technical complexity**						
I thought making the videos was easy	0 (0.0)	4 (4.4)	31 (34.4)	34 (37.8)	21 (23.3)	4 (1)
The instructions for making the videos were easy to understand	0 (0.0)	0 (0.0)	1 (1.1)	29 (32.2)	60 (66.7)	5 (1)
The instructions for accessing the videos once they were made were easy to understand	0 (0.0)	0 (0.0)	2 (2.2)	35 (38.9)	53 (58.9)	5 (1)
**Experience and satisfaction**						
Making the videos motivated me to learn about mental health problems	0 (0.0)	0 (0.0)	7 (7.8)	37 (41.1)	46 (51.1)	5 (1)
I think that making the videos was a very positive experience	0 (0.0)	0 (0.0)	2 (2.2)	28 (31.1)	60 (66.7)	5 (1)
I would like the videos to be used more often in the teaching of clinical skills	0 (0.0)	0 (0.0)	7 (7.8)	40 (44.4)	43 (47.8)	4 (1)
**Usefulness in improving knowledge and clinical skills**						
Being able to watch the videos at any time is very convenient and useful when it comes to improving knowledge on the subject	0 (0.0)	0 (0.0)	10 (11.1)	41 (45.6)	39 (43.3))	4 (1)
I think that making the videos improved my acquisition of mental health knowledge	0 (0.0)	0 (0.0)	9 (10.0)	47 (52.2)	34 (30.0)	4 (1)
I have learnt clinical skills through using the videos	0 (0.0)	7 (7.8)	22 (24.4)	34 (37.8)	27 (14.4)	4 (1)
After making the videos, I feel more prepared for clinical practice	0 (0.0)	6 (6.7)	40 (44.4)	31 (34.4)	13 (14.4)	3 (1)
I think I will use the videos in the future to review clinical skills	0 (0.0)	6 (6.7)	40 (44.4)	24 (26.7)	20 (22.2)	3 (1)
**Reduction of stigma**						
After watching the videos, I have more empathy for people with mental health problems	0 (0.0)	0 (0.0)	8 (8.9)	38.9 (26.7)	47 (52.2)	5 (1)
After watching the videos, I am no longer afraid of dealing with people with mental health problems	0 (0.0)	1 (1.1)	30 (33.3)	35 (38.9)	24 (26.7)	4 (2)
